# Occurrence of African cassava mosaic virus (ACMV) and East African cassava mosaic virus – Uganda (EACMV-UG) in *Jatropha curcas*

**DOI:** 10.1186/1753-6561-5-S7-P93

**Published:** 2011-09-13

**Authors:** Rose Ramkat, Alberto Calari, Fatemeh Maghuly, Margit Laimer

**Affiliations:** 1 Plant Biotechnology Unit (PBU), Dept. Biotechnology, University of Natural Resources and Life Sciences, Muthgasse 18, 1190 Vienna, Austria

## Background

*Jatropha curcas* is a drought resistant shrub native in tropical America, now widely grown in many tropical and subtropical regions for biodiesel production [1]. First reports on virus infections in *Jatropha* indicated the occurrence of viruses closely related to *Cassava mosaic virus* in India, reaching a disease incidence from 25 to 47%. This might represent a major constrain to the production of *Jatropha* in large scale [2]. The genome of *Cassava mosaic geminiviruses* (CMG) consist of two components termed DNA A and DNA B (~ 2.7 – 3.0 kb) [3]. Furthermore, *Jatropha* has been described as host of *Cucumber mosaic virus* (CMV) [2].

## Methods

In this study we attempted to detect and molecularly characterize viruses infecting *Jatropha* in Eastern Africa (Kenya and Ethiopia). Detection methods will be valuable tools for early screening of plant viruses in order to make appropriate decisions and selection of planting material.

A total of 127 *Jatropha* samples from Ethiopia and Kenya (districts: Kakamega, Siaya, Busia and Nakuru showing typical virus symptoms and symptomless plants were used in this study. ELISA was performed to detect the presence of three RNA viruses: CMV, *Cassava common mosaic virus* (CsCMV) and *Cassava brown streak virus* (CBSV). PCR was performed using newly designed primers based on multiple alignments of full length DNA A sequences of geminiviruses available in the NCBI Genbank, reported to infect either *Jatropha* or cassava. This allowed to amplify the variable regions of full length (2800 bp) and shorter sequences (380-1085 bp). PCR products were sequenced. A phylogenetic tree was constructed from multiple alignments by performing a heuristic search. Multiple alignments were analyzed by maximum parsimony with full-length DNA A using Phylogenetic Analysis Using Parsimony (PAUP) and a bootstrap analysis with 1000 replicates.

## Results and conclusions

None of the *Jatropha* samples analysed was infected with the RNA viruses CBSV, CMV and CsCMV. PCR primers amplifying a 380 bpfragment of AC1, AC2 and AC3 yielded positive results with 75% of the symptomatic samples from Kenya and further detected 20% of asymptomatic samples as positive. Furthermore, 61% of symptomatic *Jatropha* samples from Ethiopia were positive. Full length primers were able to detect 69% symptomatic *Jatropha* samples from Kenya, and also in 67% of asymptomatic samples. PCR analyses of sample K1J5 amplified the expected 2.8 kb of a near full length DNA A component of the Begomovirus sequence and an additional shorter fragment.

Complete nucleotide sequences of 34 DNA A components typical of Begomoviruses were determined in the Kenyan samples. Thirty three sequences ranged from 2770 bp to 2816 bp while one (K1J5) consisted only of 1416 bp and termed as a defective (Def) DNA. Phylogenetic analyses indicated that the defective molecule belongs to geminiviruses involved in CMG, representing a Def from DNA A of the bipartite Begomovirus ACMV. All viruses characterized in this study grouped with two previously identified Begomoviruses found in cassava in Western Kenya, namely EACMV – UG and ACMV. The Def DNA showed 96.6% sequence identity with the ACMV reference sequence [GenBank NC001467.1].

In this study, we report for the first time the detection of Begomovirus: ACMV and EACMV – UG in *Jatropha* from Kenya. From an evolutionary perspective, the phylogenetic data indicate that the virus isolates from the study were closely related to those isolated previously in Western Kenya from cassava [4,5]. Recombination and synergism that have long occurred in cassava [6,7] could have led to the recent spread of the virus in the field to infect *Jatropha*. Presence of EACMV – UG and ACMV on different *Jatropha* plants in the same field indicates the opportunity for mixed infections, hence offering good opportunities for more recombination to occur. EACMV – UG and ACMV are associated with severe synergistic epidemics on cassava. Synergism lead to a 10 - 50 fold increase in viral DNA accumulation which substantially increases the potential for a higher efficiency of vector transmission to even infect non cassava host plants [6,7]. This explain why EACMV - UG is the predominant virus in *Jatropha*. The deletions occurring in the Def DNA found in the study might affect the replication of the molecule and it might depend entirely on its helper virus for replication.

There is a possibility of *Cassava mosaic virus* in *Jatropha* being more wide spread than anticipated, since we have detected it also in *Jatropha* samples from Ethiopia.

## References

[B1] Openshaw K A review of *Jatropha curcas*: an oil plant of unfulfilled promise Biomass Energy 2000 19 1 15

[B2] Raj SK Snehi SK Kumar S Khan MS Pathre U First molecular identification of begomovirus in India that is closely related to *Cassava mosaic virus*and causes mosaic and stunting of *Jatropha curcas*L Australas Plant Dis Notes 2008 3 69 72 10.1071/DN08028

[B3] Yadava P Suyal G Mukherjee SK Begomovirus DNA replication and pathogenicity Current Science 2010 98 360 368

[B4] Bull SE Briddon RW Ssserubombwe WS Ngugi K Markham PG Stanley J Genetic diversity and phylogeography of *cassava mosaic viruses*in Kenya J Gen Virol 2006 87 3053 3065 10.1099/vir.0.82013-0 16963765

[B5] Stanley J Gay MR Nucleotide sequence of cassava latent virus DNA Nature 1983 301 260 262 10.1038/301260a0

[B6] Zhuo X Liu Y Calvert L Munoz C Otim-Nape GW Robinson DJ Harrison BD Evidence that DNA –A of a geminivirus associated with severe cassava mosaic disease in Uganda has arisen by interspecific recombination J Gen Virol 1997 78 2101 2111 9267014 10.1099/0022-1317-78-8-2101

[B7] Harrison BD Zhou X Otim-Nape GW Liu Y Robinson DJ Role of a novel type of double infection in geminivirus induced epidemic of severe cassava mosaic in Uganda Ann App Biol 1997 131 437 448 10.1111/j.1744-7348.1997.tb05171.x

[B8] Legg JP Fauquet CM Cassava mosaic geminiviruses in Africa Plant Mol Biol 2004 56 585 599 10.1007/s11103-004-1651-7 15630622

[B9] Monde G Walangululu J Winter S Bragard C Dual infection by cassava begomoviruses in two leguminous species (*Fabaceae*) in Yangambi, North Eastern Democratic Republic of Congo Arch Virol 2010 155 1865 1869 10.1007/s00705-010-0772-3 20680361

